# Mechanically Coherent Zeolite 13X/Chitosan Aerogel Beads for Effective CO_2_ Capture

**DOI:** 10.1021/acsami.1c04064

**Published:** 2021-04-26

**Authors:** Enrica Luzzi, Paolo Aprea, Martina Salzano de Luna, Domenico Caputo, Giovanni Filippone

**Affiliations:** Dipartimento di Ingegneria Chimica, dei Materiali e della Produzione Industriale (INSTM Consortium−UdR Naples), Università degli Studi di Napoli Federico II, p.le Tecchio 80, Naples 80125, Italy

**Keywords:** 13X zeolite, chitosan, CO_2_ capture, adsorption, aerogel, freeze-drying

## Abstract

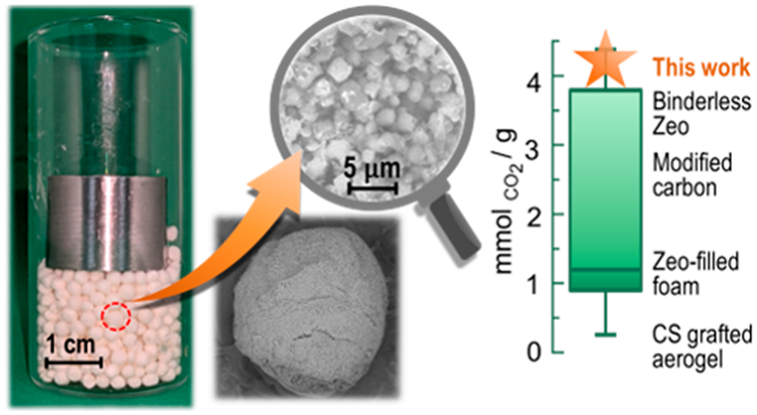

The constant increase of CO_2_ concentration in the atmosphere is recognized worldwide to severely impact the environment and human health. Zeolites possess a high adsorption capacity for CO_2_ removal, but their powdery form prevents their use in many practical applications. When binding agents are used, a partial occlusion of the porosity can severely compromise the adsorption capacity. In this regard, a great challenge is producing compact composite adsorbents while maintaining a high specific surface area to preserve the pristine performance of zeolites. Here, this goal was achieved by preparing beads with a high content of zeolite 13X (up to 90 wt %) using a chitosan aerogel as the binding agent. A facile preparation procedure based on the freeze-drying of hydrogel beads obtained by phase inversion led to a peculiar microstructure in which a very fine polymeric framework firmly embeds the zeolite particles, providing mechanical coherence and strength (compressive strain >40% without bead fragmentation, deformation <20% under 1 kg_f_-load) and yet preserving the powder porosity. This allowed us to fully exploit the potential of the constituents, reaching a high specific surface area (561 m^2^ g^–1^) and excellent CO_2_ uptake capacity (4.23 mmol g^–1^) for the sample at 90% zeolite. The beads can also be reused after being fully regenerated by means of a pressure swing protocol at room temperature.

## Introduction

CO_2_ emissions due to anthropic activities are recognized as one of the main causes of global warming. Over the past 150 years, the CO_2_ concentration in the atmosphere increased from 250 to 418 ppm,^1^ leading to critical environmental concerns. Increasing atmospheric CO_2_ concentration has been recorded even in indoor spaces, with consequent risks for human health in the case of chronic exposures.^2^ Besides regulating the emissions, new effective strategies are continuously sought to capture CO_2_ from the environment. Among the possible CO_2_ removal strategies,^3^ adsorption-based processes are receiving growing attention because of their reversibility and versatility.^4^ The working principle is based on the exploitation of an “active” material on which CO_2_ effectively adsorbs. A wide panorama of adsorbents can be used for this purpose, for example, graphene oxide,^5,6^ mesoporous silica,^7,8^ metal oxides,^9,10^ activated carbons,^11,12^ metal-organic frameworks (MOFs),^13,14^ and zeolites.^15−18^ The latter are particularly suitable for fast and reversible CO_2_ adsorption devices because of good selectivity toward CO_2_ at low pressures and moderate temperatures^18^ and the possibility to be fully regenerated with minimal energy consumption (e.g., by pressure- or temperature-swing adsorption).^19,20^ The main technological drawback of zeolites is that they are commonly synthesized in a powdery form, while monolithic adsorbents such as pellets and beads are preferred for large-scale applications. Among the possible strategies to obtain coherent zeolite-based adsorbents,^21−23^ the simplest one is embedding powders in a composite structure by means of a binding agent. In such cases, the challenge is preserving the zeolite porosity without compromising its CO_2_ capture ability.^25^ As an example, Wu et al. successfully developed a self-standing composite with an open porous structure by dispersing powdery zeolitic imidazolate frameworks in a polyimide.^26^ Nevertheless, low CO_2_ adsorption capacity was recorded (0.446 mmol g^–1^) because of the collapse of the organometallic frameworks during aerogel preparations. Valencia et al. prepared a silicalite high-loaded hybrid foam with high mechanical stability.^23^ In this case, the relatively low CO_2_ capture ability (1.3 mmol g^–1^) was ascribed to a partial shielding of the active phase embedded in the matrix. While leading to relatively low performance, the previous studies indicate the direction one can take for maximizing the CO_2_ capture capacity of zeolite-based adsorbents: (i) working at a high zeolite content to maximize the amount of the active phase; (ii) using a highly porous matrix as a binding agent not to occlude the zeolite porosity and to allow the CO_2_ to reach the entire active surface. For this purpose, we embedded zeolite 13X (ZX) in a chitosan (CS) aerogel. The amino groups of CS, which is a biopolymer derived from chitin,^27^ could enhance the CO_2_ capture ability of ZX due to possible acid–base interaction with CO_2_.^28^ More importantly, CS allows one to obtain highly porous aerogels by simple freeze casting of aqueous solutions. In this way, millimetric ZX/CS aerogel beads at very high ZX content were easily and safely prepared and fully characterized in terms of textural properties, CO_2_ adsorption performances, and mechanical properties. Besides possessing remarkable CO_2_ adsorption capacity, the beads were coherent, mechanically stable, and reusable after regeneration with a mild pressure swing protocol.

## Experimental Section

### Materials

Chitosan powder (deacetylation degree 77%, average molecular weight of 200–300 kDa) and zeolite 13X powder were purchased form Sigma-Aldrich and used without further purification.

### Preparation of CS-ZX Aerogel Beads

**Figure 1:**
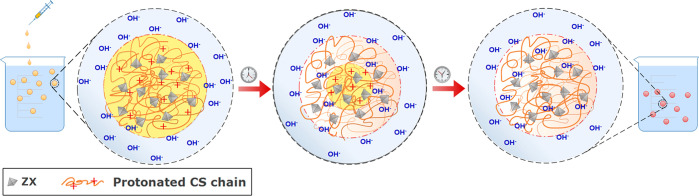
Schematic representation of the hydrogel beads formation by the phase inversion process.

CS-ZX aerogel beads at different zeolite concentrations (φ_ZX_ = ZX/CS w/w) were prepared by freeze-drying of hydrogel beads obtained by phase inversion. In detail, CS powder was added at a concentration of 10 mg/mL to a 2 vol % solution of acetic acid in distilled water under stirring at room temperature. After complete dissolution of the polymer, the pH was increased up to ∼5.5 by adding a proper amount of 5 M NaOH solution. This step is necessary since ZX is unstable in acidic conditions (see Section S1 in the Supporting Information). Then the zeolite powder was added under vigorous stirring until a homogeneous whitish dispersion was obtained. The hydrogels beads were prepared by the phase inversion method (see Figure 1). Briefly, the CS-ZX dispersion was added drop by drop with a syringe to an alkaline bath (0.05 M NaOH). The diffusion of the OH^–^ ions from the solution to the core of the drops causes the formation of physical junctions among CS chains, and the CS-ZX dispersion drops turned into hydrogel beads embedding the ZX powder. After being washed with water to remove sodium acetate possibly formed during the process (see Section S1 in the Supporting Information), the beads were frozen by immersion in hexane at −25 °C and finally freeze-dried to obtain CS-ZX aerogel beads.

### Characterization

The size distribution of the aerogel beads was obtained by image analysis (ImageJ)^29^ of photographs of the samples. An equivalent radius (*r*_EQ_) was defined for each bead as the radius of the circle having the same area as the bead projection in the plane of the picture. The surface texture and inner microstructure of razor-blade-cut samples were investigated by scanning electron microscopy (SEM) with a TM3000 Tabletop Microscope. The mechanical behavior of the aerogel beads was investigated by confined uniaxial compression tests (Tensometer 2020) carried out at 10 mm/min on a fixed volume beads column (∼5 cm^3^). The specific surface area (SSA) was measured by N_2_ adsorption/desorption isotherms at −196 °C with a Micromeritics ASAP 2020 instrument, using the Brunauer–Emmett–Teller (BET) method. The CO_2_ capture ability was evaluated by performing adsorption isotherms at 25 °C with a custom gravimetric apparatus based on a McBain-type balance.^30^ Briefly, the sample was placed in a pan attached to a silica glass spring in a glassy adsorption chamber. The samples were activated by degassing the chamber at 150 °C for 2 h under a high vacuum. The sample stability at this temperature was previously assessed by thermogravimetric analysis (see Section S2 in the Supporting Information). The sample mass was derived from the spring displacement, which increased as CO_2_ feeds the chamber because of gas adsorption. Experiments were repeated three times on samples previously degassed at 150 °C for 2 h under high vacuum. Selectivity was estimated by measuring the N_2_ adsorption isotherms in the same conditions as CO_2_ adsorption experiments. The process reversibility and the possibility of reusing the CS-ZX aerogel beads were evaluated by performing kinetic tests at 25 °C conducted with the same McBain-type balance used for the CO_2_ adsorption experiments. Briefly, the CO_2_ pressure was manually switched between 1 bar and vacuum over four adsorption/desorption cycles.

## Results and Discussion

### Morphology, Microstructure, and Porosity of the Aerogel Beads

The preparation protocol resulted in pretty spherical beads (circularity *c* = 4π(area/perimeter^2^) > 0.6) with nearly symmetric size distributions irrespective of ZX content (see Section S3 in the Supporting Information). The average *r*_EQ_ slightly increases with φ_ZX_, ranging from ∼1.2 mm (pure CS) to ∼1.4 mm (CS-ZX sample at φ_ZX_ = 0.9). This is likely due to a stabilizing action of the zeolite particles, which reduce bead shrinking during hydrogel formation.

**Figure 2:**
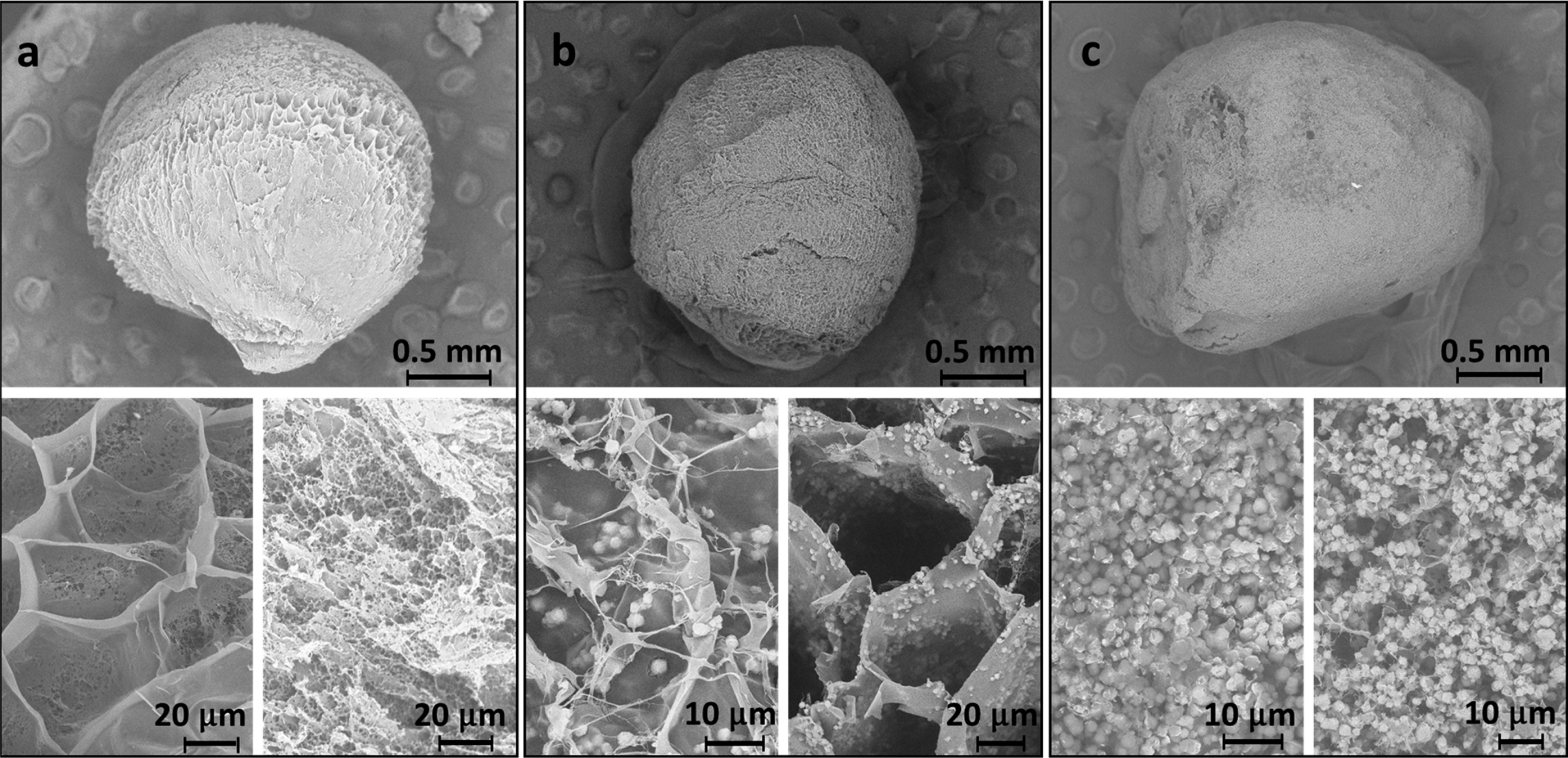
SEM micrographs of aerogel beads: (a) CS, (b) CS-ZX at φ_ZX_ = 0.5, and (c) CS-ZX at φ_ZX_ = 0.9. In each panel, bottom images show the external surface (left) and the inner section (right) of the bead at higher magnification.

The surface and internal microstructure of the beads are shown in Figure 2, where SEM micrographs are reported for pure CS and two CS-ZX samples at different φ_ZX_. The external surface of the pristine CS bead exhibits the typical morphology of CS aerogels,^31^ characterized by a semicontinuous skin with irregular cavities through which the microsized interconnected porosity of the bulk can be guessed (Figure 2a). The inner morphology of the beads significantly changes in the CS-ZX sample at φ_ZX_ = 0.5, evolving in a honeycomb-like structure, in which CS walls embed homogeneously dispersed ZX particles (Figure 2b). An analogous morphological transition has been observed by Wu et al. upon the addition of ZIF-8 particles in polyimide-based aerogels.^26^ A drastic change in the bead morphology is noticed in the sample at φ_ZX_ = 0.9 (Figure 2c). The surface acquires a grainy texture, reminiscent of that of binder-free ZX monoliths obtained by Wang et al. with a complex three-step procedure involving 3D printing, calcination, and particle soldering induced by hydrothermal crystallization.^32^

**Figure 3:**
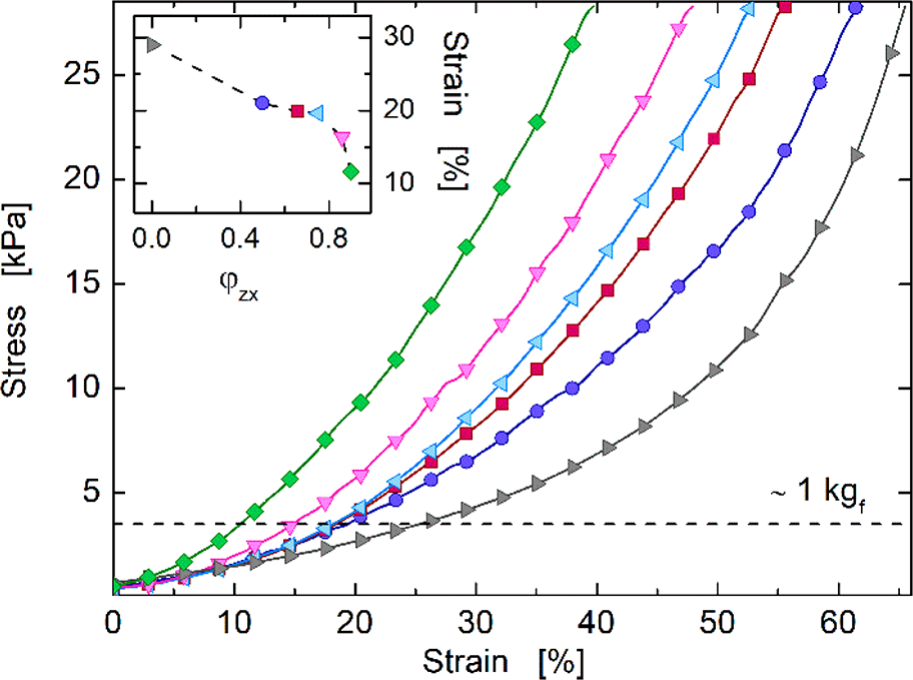
Stress–strain curves of an aerogel beads column under confined uniaxial compression. The inset shows the column deformation for 1 kgf; symbols are the same as those in the main plot.

Despite their grainy texture, the beads are mechanically coherent and strong. This is shown in Figure 3, where the results of uniaxial confined compression tests are reported. The column of beads bears increasing stresses while densifying, reaching compressive strain >40% without bead fragmentation. Taking an applied load of 1 kg_f_ as reference, the deformation decreases as the zeolite content is increased (Figure 3, inset).

**Figure 4:**
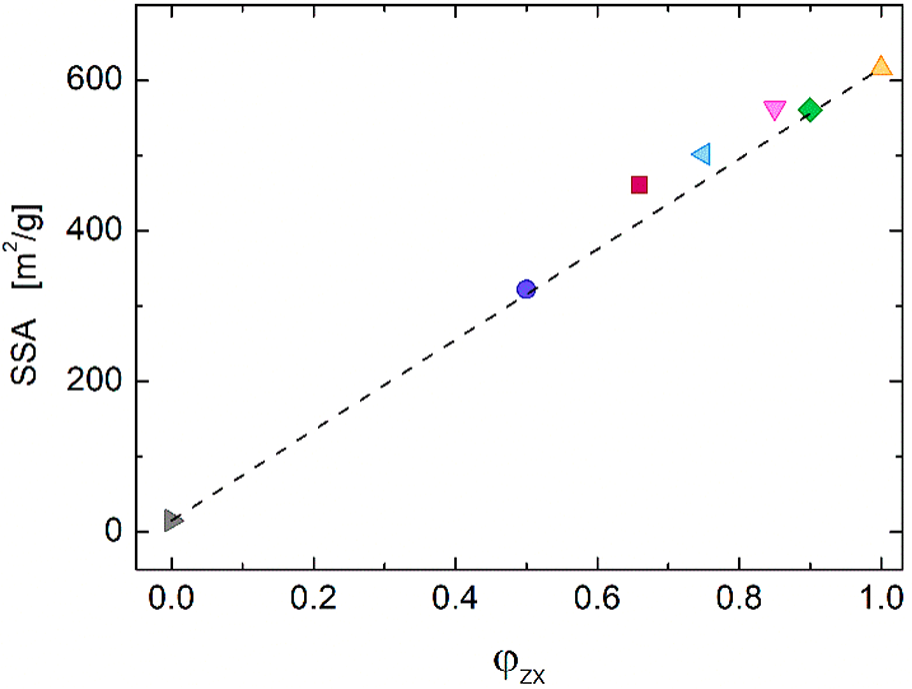
Specific surface area measured with the BET method. Dashed line shows the expectations of the mixing rule.

Besides providing mechanical coherence and strength, an ideal binding agent must not occlude the porosity of the active powder to preserve the accessibility to its whole surface area.^23^ For verification that the ZX particles were fully exposed and accessible in all CS-ZX samples, N_2_ adsorption/desorption isotherms were performed to evaluate the specific surface area (Figure 4). The SSAs of pure CS aerogel (not shown) and ZX powder are ∼15 and ∼616 m^2^ g^–1^, respectively. The data of the CS-ZX composite aerogels fall between these two extremes, with a small positive deviation from the mixing rule (dashed line in Figure 4). This proves that zeolite particles do not suffer from pore occlusion when embedded in the CS matrix, rather benefiting from an optimized surface exposure due to excellent dispersion.

### CO_2_ Adsorption Ability

**Figure 5:**
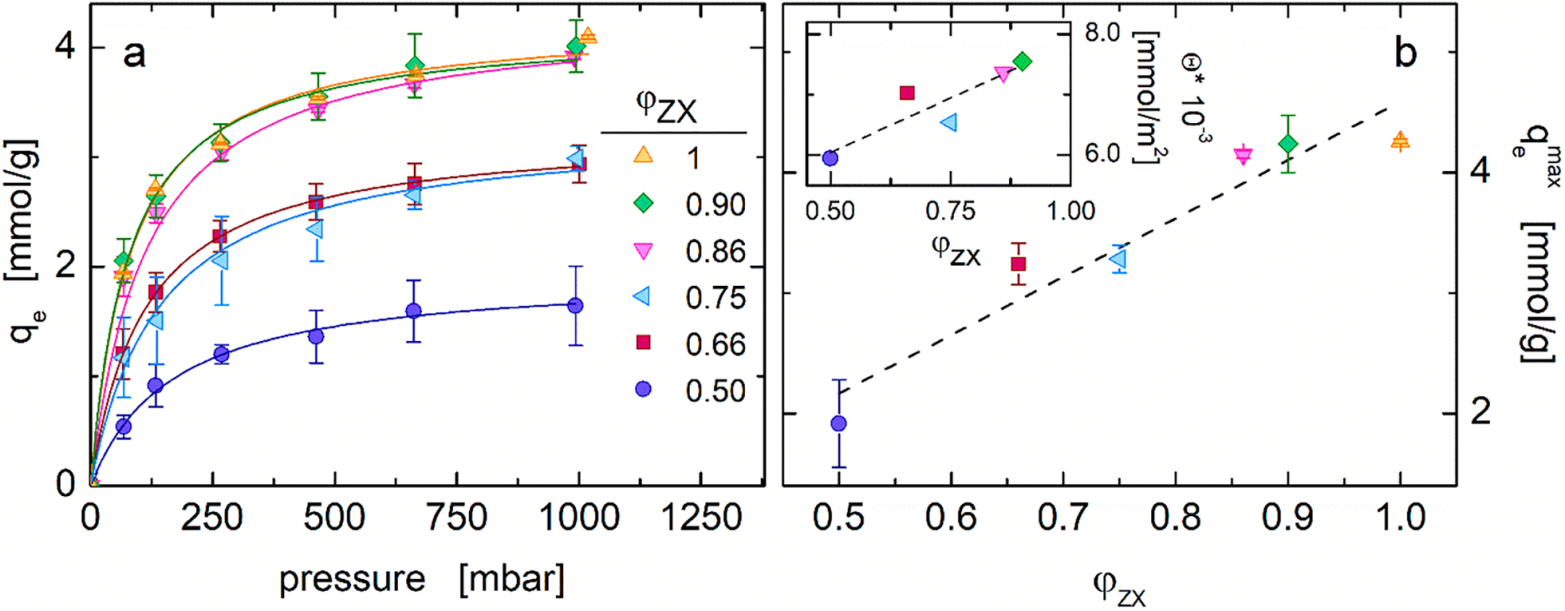
(a) CO_2_ adsorption isotherms of CS-ZX aerogel beads. Solid lines represent the best fitting of the Langmuir model to experimental data. (b) Influence of the zeolite amount on the maximum adsorption capacity; same symbols as in panel (a). The inset shows the adsorption capacity normalized to the specific surface area as a function of zeolite content.

The CO_2_ isotherms for CS-ZX aerogels are reported in Figure 5a, in which the adsorbed CO_2_ amount (*q*_e_) is plotted as a function of the gas pressure at equilibrium. Since the CO_2_ adsorption capacity of the pure CS aerogel is below instrument resolution, the remarkable CO_2_ uptake of the CS-ZX samples can be primarily ascribed to zeolite, and in fact increases with φ_ZX_. The behavior of the investigated systems is satisfactorily described by the Langmuir model^33^ (see Section S4 in the Supporting Information). The maximum CO_2_ adsorption capacity (*q*_e_^max^) linearly grows with φ_ZX_, suggesting a direct relationship between CO_2_ adsorption capacity and available SSA.

**Figure 6:**
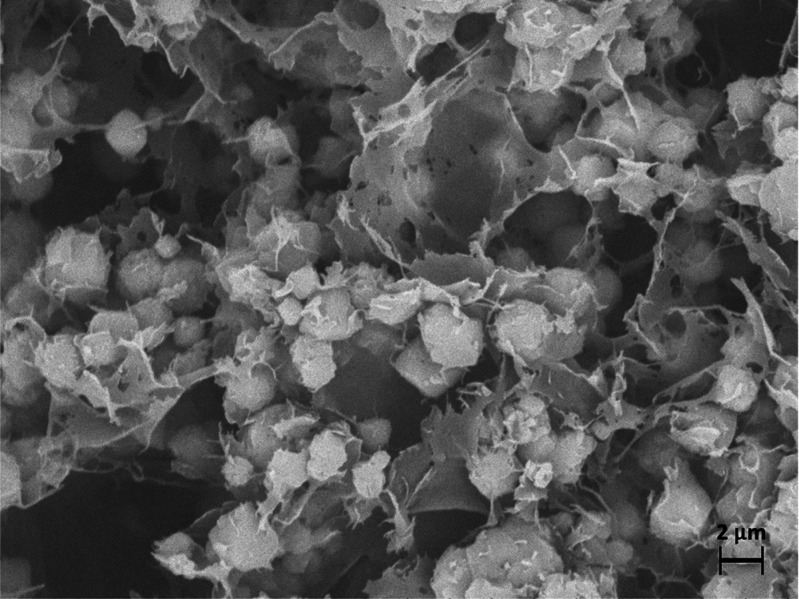
SEM micrograph of the bead section for the aerogel at φ_ZX_ = 0.9.

Actually, the adsorption capacity divided by the specific surface area (Θ = *q*_e_^max^ SSA^–1^) increases with φ_ZX_ (see Figure 4b, inset). This growing trend, not observed in similar composite aerogels made of an active material in an inert binder,^23^ indicates that CS may play an active role in CO_2_ adsorption. Moreover, the increase of Θ with φ_ZX_ indicates that this role of CS especially emerges when its content is very low. When properly exposed to the adsorbate, the amino groups of CS chains are known to react with CO_2_ according to the following mechanism:^34^
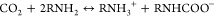
1Such a reaction could be promoted by the peculiar microstructure achieved in our composite aerogels at high zeolite content. The high-magnification SEM micrograph of Figure 6 highlights the state of the CS in the sample at the highest φ_ZX_. The polymer appears in the form of thin (∼10^2^ nm) films and fibrils that bind the zeolite particles. Such a confined conformation is likely to maximize the exposure of amino groups of CS, providing the binding agent with inherent adsorption activity toward CO_2_ that adds to that of zeolite. A similar mechanism has been proposed for nanoporous polyethylenimine-silica monoliths,^34,7^ and it has been observed in SiO_2_ nanoparticles coated by chitosan films.^28^ Here, the synergic effect of the aerogel constituents and the optimal microstructure of the beads result in remarkable CO_2_ adsorption performances. This clearly emerges from Table 1, which shows literature data of CO_2_ uptake for different chitosan- and/or zeolite-based adsorbents specifically designed for CO_2_ removal. Besides being close to that of pure ZX powder (4.26 mmol g^–1^), the CO_2_ capture capacity of the CS-ZX sample at φ_ZX_ = 0.9 is among the highest values reported to date for comparable adsorbents, including chitosan- and/or zeolite-based materials. Finally, the beads also exhibit selectivity toward the CO_2_ with respect to N_2_. In particular, the CO_2_-*q*_e_^max^ is always higher than N_2_-*q*_e_^max^, with a selectivity factor of 10.4 (see Section S5 in the Supporting Information).

**Table 1:** CO_2_ Uptake Capacity (25 °C, 1 bar) of Different Adsorbents (Data Taken from the Literature)

adsorbent	*q*_e_^max^ [mmol g^–1^]
chitosan-grafted graphene oxide aerogel^24^	0.257
polyimide/ZIF-67^26^	0.446
carbonized chitosan^35^	0.45
nanocellulose/ZIF-based foams^36^	0.75
polyethylenimine-functionalized SBA-15^7^	0.81
graphene/ZIF-8 aerogel^13^	0.99
ZIF-based nanosheets^37^	1.036
zeolite Y-chitosan composite^38^	1.098
ZIF/graphene oxide based nanocomposites^39^	1.1
ethylenediamine/graphene oxide aerogel^40^	1.18
microporous carbon spheres^41^	1.2
silicalite/cellulose foams^23^	1.3
amidoxime-modified porous carbon^42^	2.871
Binderless zeolite monoliths^21^	3.7
MOF nanofibrous membrane^43^	3.9
chitosan-graphene oxide aerogels^5^	4.15
**CS-ZX aerogel beads at φ**_**ZX**_**= 0.9**^**This work**^	**4.23**
chitosan-SiO_2_ mesoporous nanoparticles^28^	4.39

### Aerogels Reusability

**Figure 7:**
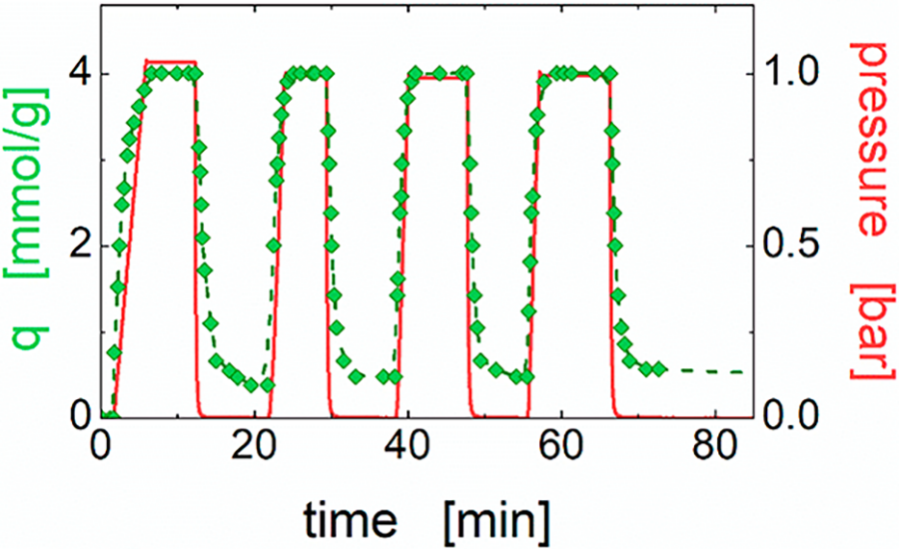
Kinetic CO_2_ adsorption/desorption isotherm performed on the sample at φ_ZX_ = 0.9.

Besides ensuring a high uptake capacity, an effective adsorbent should be fully regenerable and reusable without losing its performance.^4^ Temperature and/or pressure vacuum swing adsorption are particularly suited for zeolitic adsorbents.^44^ As emerged during data collection to produce the CO_2_ adsorption isotherms of Figure 5, a treatment at 150 °C for 2 h under high vacuum is sufficient to fully restore the weight and CO_2_ uptake performance of our samples. The effectiveness of milder regeneration conditions was also investigated for the sample at φ_ZX_ = 0.9, which was subjected to vacuum treatment at room temperature. The time dependency of the CO_2_ uptake (*q*_t_) was monitored over four adsorption/desorption cycles, and it is reported in Figure 7 together with the pressure profile. The kinetic experiment confirms that the overall adsorption capacity is not affected by repeated regenerations, and its value of about 4 mmol g^–1^ remains close to that obtained under static conditions. The adsorption/desorption kinetics is very fast, closely following the pressure profile. A small deviation is observed only at the end of the desorption steps when the CO_2_ loss slows down and eventually stops as the pressure falls below ∼0.2 bar. A residual CO_2_ quantity of about 0.5 mmol g^–1^ is not desorbed, at least in the investigated time window of about 10 min. Since pristine zeolite fully desorbs CO_2_,^45^ this residue is ascribed to chitosan, whose amino groups could form acid–base interactions with the CO_2_ molecules, which are stable at room temperature.^28^ Although the net CO_2_ uptake capacity of the material remains competitive even after a room temperature vacuum desorption, it is worth noting that a relatively low temperature treatment fully restores the excellent CO_2_ adsorption performance of our CS-ZX aerogel beads.

## Conclusions

Zeolite 13X powder was successfully embedded in a chitosan framework to obtain composite aerogel beads by a phase-inversion method followed by freeze-drying. The beads exhibited excellent CO_2_ uptake capacity owing to an optimal dispersion of the zeolite powder, whose inherent specific surface area was preserved, even at very high loadings (561 m^2^ g^–1^ at 90 wt %). Besides providing mechanical coherence, chitosan likely played an active role in CO_2_ capture when present at low concentration.

This can be due to an enhanced exposure of the amino groups that occurs when the polymer is highly confined among zeolite microparticles. The possible synergic effect of the constituents and the optimized microstructure of the beads resulted in excellent CO_2_ capture ability, reaching 4.23 mmol g^–1^ in the sample at 90 wt % zeolite loading. Finally, the possibility of reusing the aerogels over repeated adsorption/desorption cycles was also proved by means of a pressure-swing-based regeneration protocol.

## Supporting Information Available

X-ray diffraction analyses, thermogravimetric analysis, size distribution and bead column density data, Langmuir model best fitting parameters, and gas adsorption selectivity (PDF)
