# Supplementary material for: Mechanically Coherent Zeolite 13X/Chitosan Aerogel Beads for Effective CO2 Capture
Source: ACS Appl Mater Interfaces. 2021 Apr 26;13(17):20728–34. doi: 10.1021/acsami.1c04064 (PMC8289193; doi:10.1021/acsami.1c04064)
Supplement: Supplementary file 1 — am1c04064_si_001.pdf [file am1c04064_si_001.pdf]

SUPPORTING INFORMATION

Mechanically coherent zeolite 13X/chitosan aerogel beads for effective CO_2 capture

Enrica Luzzi, Paolo Aprea*, Martina Salzano de Luna*, Domenico Caputo, Giovanni Filippone

Dipartimento di Ingegneria Chimica, dei Materiali e della Produzione Industriale (INSTM Consortium−UdR Naples) - Università degli Studi di Napoli Federico II, P.le Tecchio 80, 80125 Naples, Italy.

TABLE OF CONTENTS

S1. X-Ray diffraction analyses

S2. Thermogravimetric analysis (TGA)

S3. Size distribution and bead column density data

S4. Langmuir model parameters

S5. Gas adsorption selectivity

S1. X-Ray diffraction analyses

XRD analyses were carried out using a X'Pert Pro X-ray diffractometer (Malvern Panalytical), equipped with a PIXCel 1D detector. The 2θ scan range was 5-60° with a step size of 0.01°. The XRD patterns of CS/ZX aerogel beads obtained following different procedures are reported in Figure S1 and S2. In particular, Figure S1 proves that a strong acidic environment (as the initial CS solution is, pH ~ 3.5) causes dissolution of ZX powder and hence the loss of its crystalline structure. Then, further XRD analysis highlights that washing the hydrogel beads obtained by the "phase inversion" method is necessary to remove sodium acetate, whose formation is proved from its characteristic peaks identified in Figure S2.

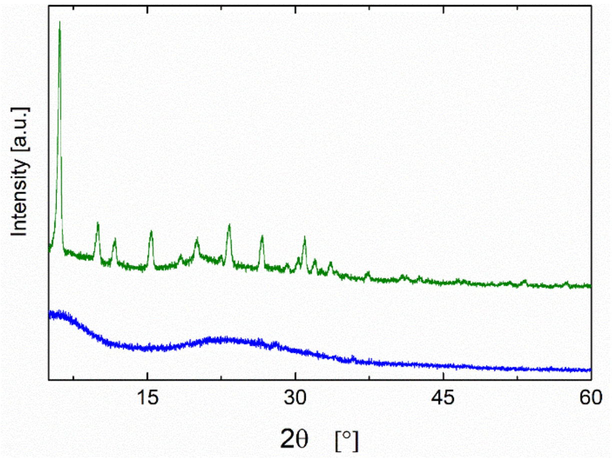

Figure S1. XRD patterns of CS/ZX aerogel beads at φ_{ZX} =0.50 obtained with (green line) or without (blue line) pH adjustment to 5.5.

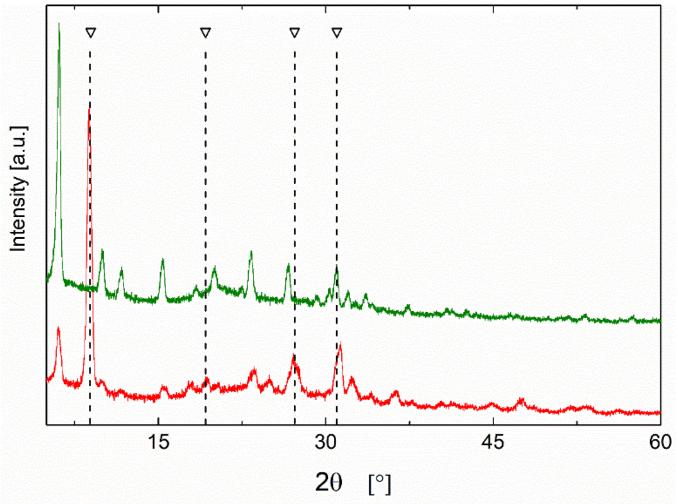

Figure S2. XRD patterns of CS/ZX aerogel beads at φ_{ZX} =0.50, obtained from non-washed (red line) or washed (green line) samples. Peaks at 2θ= 8.88°, 19.238°, 27.208° and 30.95° are characteristic of sodium acetate

S2. Thermogravimetric analysis (TGA)

Thermogravimetric analyses were carried out using a TGA Q500 (TA Instruments) with a heating rate of 20 °C min^{-1} in air atmosphere from 150°C to 700 °C. The samples were previously dried at 150°C for 30 minutes. Figure S3 shows the thermogravimetric (TG) and derivative (DTG) curves of CS-ZX aerogel beads for all the investigated compositions. The analyses reveal a good correlation between nominal and measured zeolite content (see inset of Fig. S3). Moreover, the thermal stability of chitosan increases in the presence of zeolite likely due to a shielding effect of the latter, passing from ~270°C (pure chitosan) to ~298°C at φ_{ZX}=0.9 (see Figure S3b).

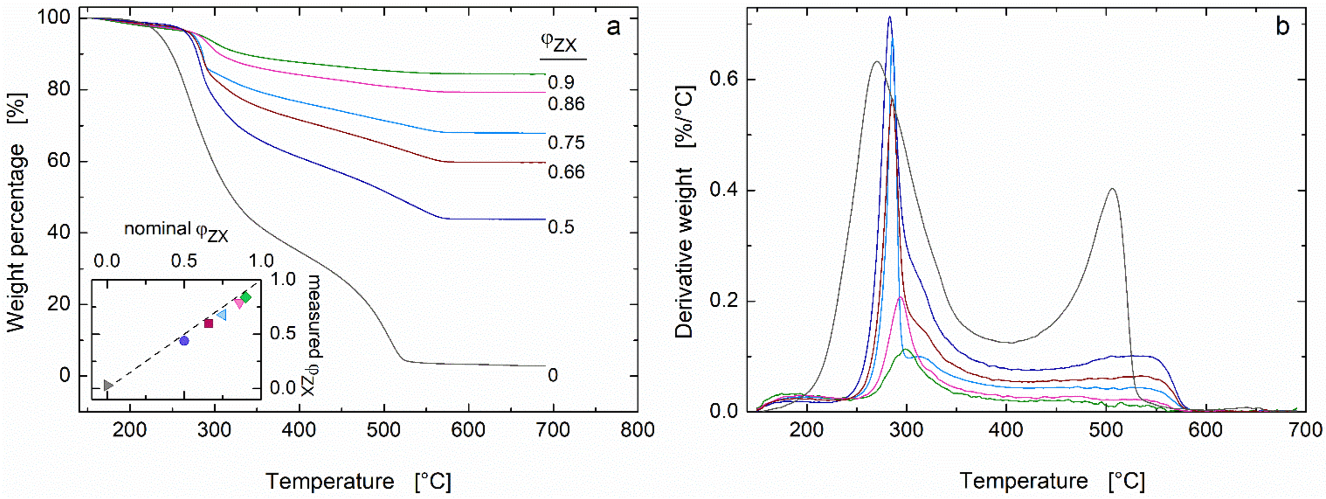

Figure S3. a) TG and b) DTG curves of CS-ZX aerogel beads; same colors as in panel a). The inset shows the φ_{ZX} values measured through TGA analyses vs the nominal φ_{ZX} values.

S3. Size distribution and bead column density data

The bead size distributions of the samples not shown in the main text are reported in Figure S4. The average bead radius (r_{EQ}) of all the investigated CS/ZX aerogels is reported in Table S1, together with the density of a loosely packed beads column (ρ_c), obtained from the weight of the column of beads put in a graduated cylinder.

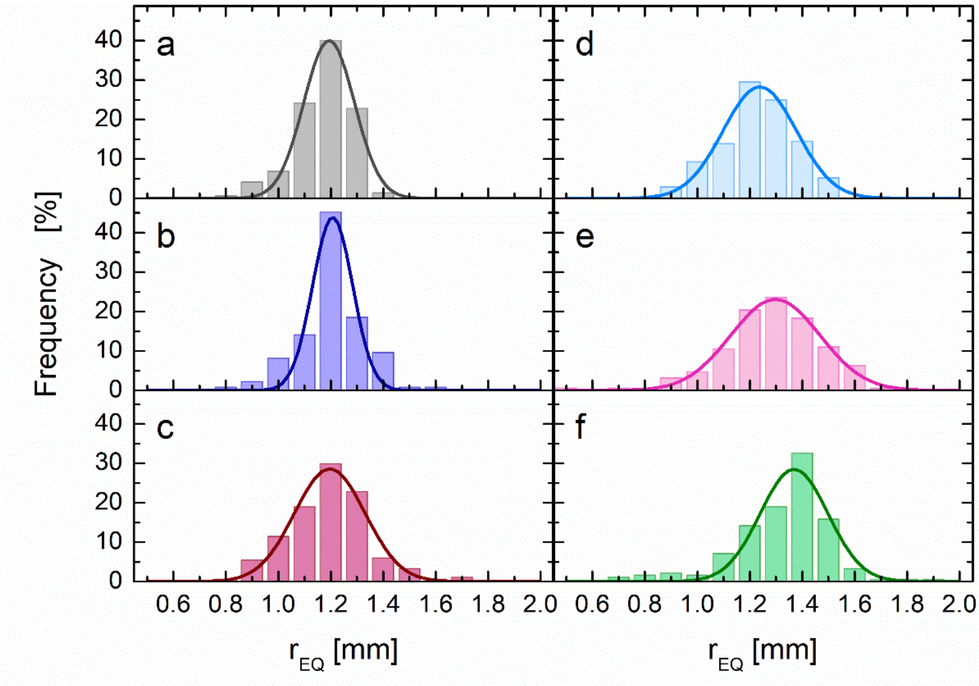

Figure S4. Aerogel beads size distribution for CS/ZX samples at φ_{ZX} = a) 0, b) 0.50, c) 0.67, d) 0.75, e) 0.86, and c) 0.90. Lines are guides for the eye.

Table S1. Average size of the beads and bead column density values for all the investigated aerogel compositions.

φ_{zeo}	r_{EQ} [mm]	ρ_c [mg cm^{-3}]
0 (pristine CS)	1.22±0.10	24.4±0.4
0.50	1.25±0.12	34.6±1.1
0.67	1.24±0.15	53.9±0.1
0.75	1.28±0.14	70.9±1.0
0.86	1.34±0.17	126.6±3.0
0.90	1.38±0.18	149.8±0.6

S4. Langmuir model parameters

The isotherms in Figure 5a of the main text were fitted to the Langmuir model: $q_e = q_e^{max} \frac{bp}{1+bp}$, in which p is the equilibrium pressure, q_e^{max} is the maximum adsorption capacity, and b is the affinity constant. The obtained best fitting parameters are reported in Table S2.

Table S2. Langmuir model parameters for all the investigated systems.

φ_{ZX}	q_e^{max} [mmol g^{-1}]	b [bar^{-1}]
0.50	1.92 ± 0.06	6.2·10-3 ± 6·10-4
0.66	3.24 ± 0.02	8.9·10-3 ± 2·10-4
0.75	3.28 ± 0.14	6.6·10-3 ± 1.0·10-3
0.86	4.15 ± 0.08	1.15·10-2 ± 9·10-4
0.90	4.23 ± 0.08	1.26·10-2 ± 1.1·10-3
1	4.26 ± 0.10	1.22·10-2 ± 1.2·10-3

S5. Gas adsorption selectivity

Comparison between CO_2 and N_2 adsorption isotherms performed on the sample at φ_{ZX} = 0.9 are reported in figure S5. In particular, the adsorption selectivity factor for CO_2 over nitrogen ($S_{CO2/N2}$), was calculated as reported from Principe et al. (*Adsorption (2020) 26:723–735)* for pure gas pressures of 1 atm.

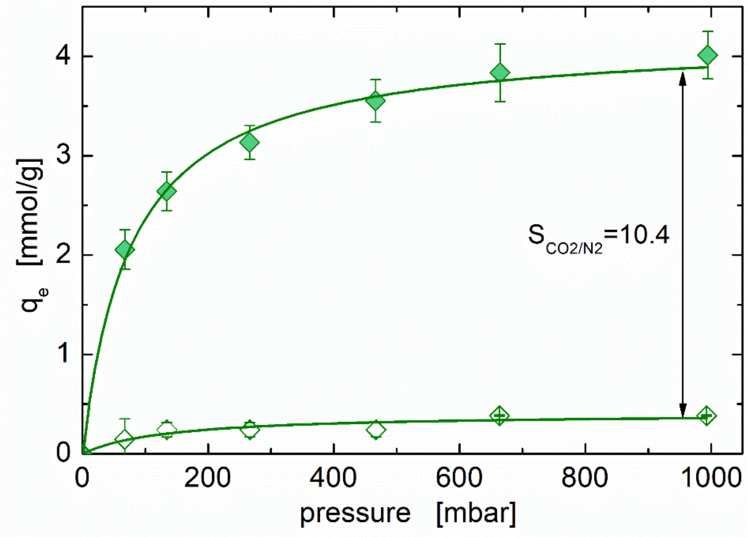

Figure S5. CO_2 (full symbols) and N_2 (empty symbols) adsorption isotherms for CS/ZX samples at φ_{ZX} = 0.90.
